# Chronic non-communicable diseases and COVID-19: EPICOVID-19 Brazil results

**DOI:** 10.11606/s1518-8787.2021055003673

**Published:** 2021-05-21

**Authors:** Marilia Arndt Mesenburg, Pedro Curi Hallal, Ana Maria Baptista Menezes, Aluísio J D Barros, Bernardo Lessa Horta, Fernando Celso de Barros, Fernando Pires Hartwig, Nadège Jacques, Mariangela Freitas da Silveira

**Affiliations:** I Universidade Federal de Pelotas Programa de Pós-Graduação em Epidemiologia PelotasRS Brasil Universidade Federal de Pelotas. Programa de Pós-Graduação em Epidemiologia. Pelotas, RS, Brasil.; II Universidade Federal de Ciências da Saúde de Porto Alegre Departamento de Saúde Coletiva Porto AlegreRS Brasil Universidade Federal de Ciências da Saúde de Porto Alegre. Departamento de Saúde Coletiva. Porto Alegre, RS, Brasil.

**Keywords:** Coronavirus Infections, epidemiology, Noncommunicable Diseases, Socioeconomic Factors, Health Surveys

## Abstract

**OBJECTIVE:**

Describing the prevalence of chronic diseases and associated socioeconomic and demographic factors, evaluating the patterns of social distancing and the antibodies prevalence against SARS-CoV-2 and COVID-19 symptoms in carriers and non-carriers of chronic diseases.

**METHODS:**

Data from 77,075 individuals aged 20 to 59 from three steps of the EPICOVID-19 Brazil (a nationwide serological survey conducted between May and June, 2021) were assessed. The presence of antibodies against SARS-CoV-2 was examined by rapid tests. Self-reported prevalence of hypertension, diabetes, asthma, cancer, chronic kidney disease and heart disease were investigated. The prevalence of mask use, adherence to isolation measures and antibodies were evaluated separately amid carriers and non-carriers of chronic diseases. The prevalence of symptoms was analyzed among carriers and non-carriers of chronic diseases with antibodies.

**RESULTS:**

The prevalence of at least one chronic disease was 43%, higher in the Southeast region, among white and indigenous individuals, women, less schooled and in lower socioeconomic position. The use of masks when leaving home was similar among carriers and non-carriers of chronic diseases (98%). The proportion of participants who reported adherence to isolation measures was higher amid carriers (15.9%) than non-carriers (24.9%) of chronic diseases. The prevalence of antibodies to SARS-CoV-2 was similar amongst carriers and non-carriers (2.4% and 2.3%). The prevalence of cough, dyspnea, palpitations and myalgia was significantly higher among carriers, but the proportion of symptomatic patients was similar between groups.

**CONCLUSION:**

The prevalence of chronic diseases in Brazil is high and the COVID-19 pandemic affects carriers and non-carriers of chronic diseases similarly. Carriers present more severe forms of COVID-19 and higher prevalence of symptoms. Greater adherence to social distancing measures among chronic patients is disassociated from a lower incidence of COVID-19 in this group.

## INTRODUCTION

By early April, 2021, about 132 million COVID-19 confirmed cases and 3 million deaths were accounted worldwide. The Americas and Europe comprise most of the confirmed cases. Even though Brazil accounts for approximately 3% of the global population, it comprises 10% of all cases and deaths worldwide^1^. COVID-19 mortality depends not only on the number of incident cases, but also on population factors such as age structure and comorbidity burden connected to the development of severe forms of the disease^1^.

Since the beginning of the pandemic, chronic non-communicable diseases (NCDs) such as high blood pressure, diabetes, respiratory diseases and cardiovascular diseases have been associated with the greater severity and COVID–19 lethality^2^. A meta-analysis including eight studies on the association between NCDs and COVID-19 severity showed that chronic obstructive pulmonary disease (COPD) was the strongest predictor of severe forms of COVID–19 (*odds ratio* [OR] = 6.4; 95%CI 2.4–16.9), followed by cardiovascular disease (OR = 2.7; 95%CI 1.5–4.8) and hypertension (OR = 1.9; 95%CI 1.4–2.7). Hospitalization in intensive care units was even more strongly associated with COPD, cardiovascular disease and hypertension, with OR of 17.8 (95%CI 6.5–48.2), 4.4 (95%CI 2.6–7.4) and 3.6 (95%CI 2.2–5.9)^4^, respectively. Another meta-analysis evaluated specifically the connection between diabetes and COVID-19 severity or lethality, finding a positive association (OR 2.16; 95%CI 1.74–2.68)^5^. Chronic kidney disease, cancer, asthma, liver diseases and HIV/AIDS are also related to unfavorable outcomes of COVID-19 infections^6^.

Due to the demographic and epidemiological transition in progress, Brazil displays a high load of NCDs^7, 8^. The prevalence of obesity and physical inactivity, crucial risk factors for these morbidities, is also high^8,9^. This scenario is alarming in the Brazilian COVID-19 pandemic context, as it increases the risk of severe cases in a country with an elevated incidence. Therefore, the assessment of the panorama of NCDs associated with COVID-19 infection in Brazil is fundamental. This study aims at describing the prevalence of NCDs and associated socioeconomic and demographic factors, in addition to evaluating the patterns of social distancing and the prevalence of antibodies against SARS-CoV-2 among carriers and non-carriers of NCDs, as well as the symptom occurrence among individuals with antibodies against SARS-CoV-2 carrying and not carrying NCDs.

## METHODS

We analyzed data from EPICOVID-19 Brazil, a series of national serological surveys carried out aiming at evaluating the magnitude, evolution and characteristics of the pandemic. The most populous city of each of the 133 geographical intermediate regions, according to the Brazilian Institute of Geography and Statistics (IBGE), were selected to take part in the study. Multiple stage sampling was carried out in each city, having the 2010 IBGE’s urban census sectors as primary sampling units. We selected 25 sectors with probability proportional to size in each city. In each sector, 10 households were systematically selected. In each household, a resident was drawn and invited to participate in the study. In case of refusal, a second resident was drawn. If the second resident had also refused, the interviewers would have approached the next household, immediately on the right. The same replacement procedure was adopted if the selected household was empty or did not open the door to the interviewer. All refusals and substitutions were recorded by the field team.

Each participant answered a questionnaire on socioeconomic and demographic characteristics, social distancing, mask use, presence of COVID-19 symptoms, previous diagnosis of NCDs, among others. Participants underwent the *WONDFO SARS-CoV-2 rapid Antibody Test* (Wondfo Biotech Co., Guangzhou, China), which detects the presence of antibodies against SARS-CoV-2 (IgG and IgM). The test was previously validated by the responsible research team^10,11^. The interviewers received standardized training on the questionnaire application, rapid test administration, and results analysis. All positive rapid tests and 20% of the negative ones were evaluated by a second observer. EPICOVID-19 Brazil’s detailed methodology was previously published^12^.

Data were collected in three EPICOVID-19 Brazil stages, conducted from May 14 to 21 (n = 25,025), from June 4 to 7 (n = 31,165) and from June 21 to 24 (n = 33,207), 2020. The analyses here were restricted to adults aged 20 or older.

To assess the existence of chronic diseases, patients were asked if they had ever been diagnosed with hypertension, diabetes, asthma, cancer, chronic kidney disease and heart disease (“yes” or “no”). Individuals who reported a diagnosis of one or more NCDs were considered carriers of NCDs and will be mentioned in this way throughout the text.

The presence of antibodies against SARS-CoV-2 was evaluated by the rapid test results: when the result was positive, the individual was considered to have antibodies against the virus. Mask use was assessed by the question “Do you wear a mask when you leave your house?” (“yes”, “no” or “I do not leave my house”). Adherence to social distancing was assessed by the following questions:

- “How much of the social distancing guidelines recommended by health authorities (i. e., staying at home and avoiding contact with other people) do you follow?” (“very little”, “little”, “more or less”, “quite a lot” or “mostly isolated from everyone”);
- “How has your daily routine been?” (“I stay at home all the time”, “I leave my house only for essential things, such as buying food”, “I go out from time to time to shop and stretch my legs”, “I leave my house every day for some activity”, “I go out every day, all day, to work or to do other regular activities”);
- “Thinking about the household routine, who has been attending the house?” (“only family members who live together, if any, and no one else”, “some close relatives visit once to twice a week”, “some close relatives visit almost every day”, “friends, relatives or others visit once to twice a week”, “friends, relatives or others visit almost every day”).

To assess the presence of COVID-19 symptoms, participants were asked if they had the following symptoms: fever, cough, sore throat, changes in smell or taste, myalgia, twitching, headache, breathing difficulties, palpitation, diarrhea and vomiting. Participants who reported the occurrence of at least one symptom were considered symptomatic. The recall period of questions about symptoms differed between the stages of EPICOVID-19 Brazil. The first two stages evaluated the occurrence of symptoms in the 15 days prior to the interview and the third stage evaluated the period from March 2020 to the interview date. Since antibodies against SARS-Cov-2 become detectable in the rapid test about 15 days after infection, the analysis on the occurrence of symptoms was restricted to the participants of the third stage.

The evaluated socioeconomic and demographic variables were geographic region (North, Northeast, Southeast, South or Midwest), self-reported skin color (white, black, brown, yellow or indigenous), gender (female or male), age (in categories) and schooling levels (elementary school, high school or higher education). To assess the socioeconomic position of households, the National Economic Indicator (IEN) was used. This index was created by the analysis of main components based on a set of household characteristics and goods (computer, internet access, color television, air conditioning, motor vehicles, cable television, number of rooms used for sleeping and number of bathrooms). The first component was extracted and divided into quintiles. The first quintile represents the poorest 20% and the fifth quintile, the richest 20%^13^.

The analyses included the sample description and the prevalence calculation of each NCD and the prevalence of carriers of NCDs. The prevalence of carriers of NCDs was assessed according to region, skin color, gender, age, IEN and schooling levels. The prevalence of mask use and adherence to social isolation measures, as well as the prevalence of individuals with antibodies against SARS-Cov-2, were calculated separately for carriers and non-carriers of NCDs. Finally, the prevalence of symptoms for COVID-19 between carriers and non-carriers of NCDs with antibodies against SARS-Cov-2 was calculated. For all proportions, 95% confidence intervals were calculated. We obtained p-values by a Chi-square test for heterogeneity or, for ordinal categorical variables, linear trend, considering a significance level of 5%. All analyses were conducted in Stata 16, considering the effect of sample design.

This study respected all the ethical precepts and legislation governing research with human beings and was approved by the National Commission for Research Ethics (CAAE 30721520.7.1001.5313). All participants signed the informed consent form.

## RESULTS

We analyzed EPICOVID-19 Brazil’s data from 77,075 participants aged 20 or older. Most were female (59%) and non-white (62%). About a quarter were older adults (aged 60 or older). Around 40% of the participants reported having attended elementary school (39%) and lived in the region. Considering the three stages of the study, the crude proportion of participants with antibodies against SARS-CoV-2 was 2.3% (95%CI 2.3-2.5). Circa 45% of those reported carrying one or more NCDs. The most prevalent NCDs were arterial hypertension (30%), followed by diabetes (13%), asthma (9%) and heart disease (8%) (Table 1).

**Table 1:** Sociodemographic characteristics and prevalence of chronic non-communicable diseases at the EPICOVID-19 Brazil study, stages 1 to 3 (n = 77,075).

	n	% (95%CI)
Description of sample characteristics		
Region		
North	13,075	16.8 (16.5–17.2)
Northeast	23,033	29.6 (29.1–30.1)
Southeast	19,359	25.3 (24.8–25.8)
South	13,112	17.3 (16.8–17.8)
Mid-west	8,496	11.0 (10.7–11.4)
Skin color		
White	28,387	37.8 (37.2–38.3)
Brown	33,732	44.8 (44.4–45.3)
Black	10,030	13.3 (12.9–13.6)
Yellow	2,132	2.8 (2.7–2.9)
Indigenous	1,046	1.4 (1.3–1.5)
Gender		
Male	31,239	40.5 (40.2–40.9)
Female	45,836	59.5 (59.1–59.8)
Age		
20–29	13,796	18.3 (18.0–18.6)
30–39	13,726	18.2 (17.9–18.5)
40–49	14,129	18.5 (18.2–18.8)
50–59	14,273	18.5 (18.2–18.8)
60–69	12,062	15.3 (15.1–15.6)
70–79	9,126	11.1 (10.9–11.4)
IEN (in quintiles)		
The poorest	18,106	22.7 (22.2–23.2)
2	14,161	18.2 (17.9–18.5)
3	14,748	19.3 (18.9–19.6)
4	14,909	19.7 (19.3–20.0)
The richest	15,143	20.2 (19.6–20.8)
Schooling levels		
Elementary school	30,876	39.5 (38.9–40.1)
High school	28,315	37.7 (37.3–38.1)
Higher education	17,013	22.8 (22.2–23.4)
Antibodies against SARS-CoV-2		
Yes	1,824	2.3 (2.2–2.5)
Prevalence of chronic diseases		
Hypertension	23,575	30.0 (29.6–30.3)
Diabetes	9,842	12.5 (12.2–12.7)
Asthma	7,266	9.4 (9.2–9.6)
Cancer	2,307	3.0 (2.8–3.1)
Kidney disease	3,128	4.0 (3.8–4.1)
Heart disease	6,354	8.1 (7.9–8.3)
Number of chronic diseases reported		
None	42,246	56.2 (55.8–56.6)
One	20,521	27.0 (26.6–27.3)
Two	9,164	11.9 (11.6–12.1)
Three	2,985	3.9 (3.7–4.0)
Four or more	827	1.1 (1.1–1.2)

95%CI: 95% confidence interval; IEN: Indicador Econômico Nacional (National Economic Indicator).

Table 2 shows the prevalence of one or more NCDs according to socioeconomic and demographic characteristics. The prevalence was higher in the Southeast and South (48%), among indigenous people (47%), women (47%) and individuals with lower schooling levels (57%). The prevalence of one or more NCDs presented an inverse relationship with age and quintiles of the IEN. All differences were statistically significant (p-value < 0.001).

**Table 2:** Prevalence of one or more chronic non-communicable diseases, according to socioeconomic and demographic characteristics at the EPICOVID-19 Brazil study, stages 1 to 3 (n = 77,075).

	% (95%CI)
Region	p < 0.001^a^
North	37.6 (36.7–38.4)
Northeast	42.2 (41.5–42.9)
Southeast	48.4 (47.6–49.2)
South	47.9 (46.9–48.9)
Mid-west	44.8 (43.6–45.9)
Skin color	p < 0.001^a^
White	46.9 (46.3–47.5)
Brown	41.4 (40.9–42.0)
Black	44.3 (43.2–45.3)
Yellow	42.8 (40.7–44.9)
Indigenous	47.2 (44.1–50.3)
Gender	p < 0.001^a^
Male	39.5 (38.9–40.1)
Female	47.4 (47.0–47.9)
Age	p < 0.001^b^
20–29	18.8 (18.2–19.5)
30–39	23.6 (22.9–24.3)
40–49	36.7 (35.8–37.5)
50–59	53.9 (53.1–54.8)
60–69	69.0 (68.1–69.8)
70 or older	78.3 (77.5–79.2)
IEN (in quintiles)	p < 0.001^b^
The poorest	47.7 (46.9–48.4)
2	45.3 (44.5–46.2)
3	43.3 (42.5–44.1)
4	42.1 (41.3–43.0)
The richest	42.1 (41.3–42.9)
Schooling levels	p < 0.001^b^
Elementary school	57.0 (56.4–57.6)
High school	35.6 (35.0–36.2)
Higher education	35.0 (34.2–35.7)

95%CI: 95% confidence interval; IEN: Indicador Econômico Nacional (National Economic Indicator).

^a^ Chi-square test of heterogeneity.

^b^ Linear trend test.

The results on social distancing are presented in Table 3. Around 25% of individuals carrying one or more NCDs reported being isolated from virtually everyone. Among non-carriers, this percentage was 15%. Regarding the daily activities, 13% of those with one or more NCDs reported leaving their houses every day to work, while among those without NCDs this proportion was 25%. Both among carriers and non-carriers, the proportion that reported that only the residents themselves attended the household was approximately 50%. A higher proportion of carriers with one or more NCDs reported receiving close relatives (44%) when compared to non-carriers (39%).

**Table 3:** Social distancing and mask use between carriers and non-carriers of chronic non-communicable diseases (NCDs) at the EPICOVID-19 Brazil study, stages 1 to 3 (n = 77,075).

	n	Non-carriers of NCDs	n	Carrier of one or more NCDs	p^a^
	
		% (95%CI)		% (95%CI)	
Adherence to insolation					
Very little	3,740	8.9 (8.6–9.2)	2.112	6.3 (6.1–6.6)	< 0.001
Little	4,449	10.6 (10.3–10.9)	2.621	7.9 (7.6–8.2)	
More or less	11,637	27.7 (27.2–28.1)	7.533	22.6 (22.2–23.1)	
Quite a lot	15,510	36.9 (36.4–37.4)	12.706	38.2 (37.6–38.8)	
Isolated	6,686	15.9 (15.5–16.3)	8.300	24.9 (24.5–25.4)	
Daily activities					
Stays at home all the time	4,983	11.8 (11.5–12.2)	7.930	23.7 (23.3–24.2)	< 0.001
Leaves only for essential things like buying food	19,676	46.7 (46.2–47.2)	16.482	49.3 (48.8–49.9)	
Leaves from time to time for shopping and stretch the legs	4,144	9.8 (9.5–10.1)	3.114	9.3 (9.0–9.7)	
Leaves every day for some activity	2,884	6.8 (6.6–7.1)	1.546	4.6 (4.4–4.9)	
Leaves every day. all day. to work or other regular activity	10,440	24.8 (24.3–25.3)	4.341	13.0 (12.6–13.4)	
People who attend the house					
Only family members who live together. if any. and no one else	20,903	49.9 (49.3–50.5)	15.354	46.3 (45.6–46.9)	< 0.001
Some close relatives visit once or twice a week	13,349	31.9 (31.4–32.4)	11.585	34.9 (34.4–35.5)	
Some close relatives visit almost every day	2,993	7.1 (6.9–7.4)	3.064	9.2 (8.9–9.6)	
Relatives or other people visit once to twice a week	2,895	6.9 (6.7–7.2)	1.978	6.0 (5.7–6.2)	
Relatives or other people visit almost every day	1,738	4.2 (3.9–4.4)	1.208	3.6 (3.4–3.9)	
Mask use outside the house	41,341	98.0 (97.8–98.1)	32.537	97.2 (97.0–97.3)	< 0.001

95%CI: 95% confidence interval.

^a^ Chi-square test of heterogeneity.

Except for individuals who reported a diagnosis of cancer, the differences in the prevalence of antibodies against SARS-CoV-2 between carriers and non-carriers of NCDs were minimal (Table 4). Table 5 shows the prevalence of COVID-19 symptoms among individuals with antibodies against SARS-CoV-2, both carriers and non-carriers of NCDs. Carriers of one or more NCDs reported 50% more twitching occurrences than non-carriers, in addition a to a 26% and 29% higher occurrence of coughing and dyspnea, respectively. Myalgia was 16% more prevalent among carriers of NCDs. The prevalence of the other evaluated symptoms and the proportion of asymptomatic individuals were statistically similar.

**Table 4:** Prevalence of antibodies against SARS-CoV-2 between carriers and non-carriers of chronic non-communicable diseases (NCDs) at the EPICOVID-19 Brazil study, stages 1 to 3.

NCDs	No		Yes		PR (95%CI)^a^	p
	
	n	% (95%CI)	n	% (95%CI)		
Hypertension	1,263	2.4 (2.2–2.5)	552	2.3 (2.1–2.6)	1.0 (0.9–1.1)	0.797
Diabetes	1,542	2.3 (2.2–2.4)	265	2.7 (2.4–3.0)	1.2 (1.0–1.3)	0.019
Asthma	1,664	2.4 (2.3–2.5)	154	2.1 (1.8–2.5)	0.9 (0.8–1.0)	0.148
Cancer	1,794	2.4 (2.3–2.5)	22	1.0 (0.6–1.4)	0.4 (0.3–0.6)	< 0.001
Kidney disease	1,732	2.4 (2.2–2.5)	82	2.6 (2.1–3.3)	1.1 (0.9–1.4)	0.332
Heart disease	1,710	2.4 (2.3–2.6)	101	1.6 (1.3–1.9)	0.7 (0.5–0.8)	< 0.001
One or more NCDs	1,009	2.4 (2.2–2.6)	769	2.3 (2.1–2.5)	1.0 (0.9–1.1)	0.403

95%CI: 95% confidence interval; PR: prevalence ratio.

^a^ Reference: non-carriers of chronic diseases.

**Table 5:** Prevalence of self-reported symptoms among individuals with antibodies against SARS-CoV-2 with and without chronic non-communicable diseases (NCDs) at the EPICOVID-19 Brazil study, Step 3.

Symptom	Non-carriers of NCDs		Carriers of NCDs		PR (95%CI)^a^	p^b^
	
	n	% (95%CI)	n	% (95%CI)		
Fever	242	50.8 (45.6–56.0)	196	55.2 (49.3–61.0)	0.9 (0.9–1.2)	0.212
Sore throat	174	36.6 (31.9–41.5)	124	34.9 (29.6–40.6)	1.0 (0.8–1.2)	0.629
Cough	214	45.1 (40.2–50.0)	202	56.6 (50.7–62.3)	1.3 (1.1–1.5)	0.001
Dyspnea	111	23.4 (19.4–27.8)	107	30.1 (24.8–35.9)	1.3 (1.0–1.6)	0.030
Palpitations	80	16.9 (13.4–21.1)	103	29.1 (24.0–34.8)	1.7 (1.3–2.3)	0.381
Anosmia	285	60.0 (55.0–64.8)	223	63.0 (57.3–68.3)	1.1 (0.9–1.2)	0.381
Diarrhea	137	28.8 (24.3–33.7)	108	30.3 (25.1–36.0)	1.1 (0.8–1.3)	0.645
Vomiting	36	7.6 (5.4–10.6)	36	10.1 (6.9–14.4)	1.3 (0.8–2.2)	0.203
Myalgia	233	46.9 (42.2–51.8)	192	54.4 (48.4–60.3)	1.2 (1.0–1.3)	0.034
Twitching	90	19.1 (15.2–23.7)	104	29.2 (24.3–34.7)	1.5 (1.1–2.0)	0.001
Headache	295	62.0 (57.0–66.7)	202	56.6 (50.8–62.2)	0.9 (0.8–1.0)	0.116
Symptomatic	403	86.9 (83.2–89.8)	313	90.0 (86.5–94.1)	1.1 (1.0–1.1)	0.067

95%CI: 95% confidence interval; PR: prevalence ratio.

^a^ Reference: non-carriers of chronic diseases.

^b^ Chi-square test of heterogeneity.

## DISCUSSION

Our results showed that Brazil has a high burden of chronic diseases. Despite greater adherence to social distancing measures among carriers of NCDs, the prevalence of antibodies against SARS-CoV-2 was similar between groups.

Each morbidity assessed here depicted a higher prevalence when compared to the National Health Survey results, in which 21% of participants reported a diagnosis of hypertension ^14^ (versus 30% in the present study), 6.2% of diabetes^15^ (versus 13%), 4.4% of asma^16^ (versus 9%), 1.8% of cancer^17^ (versus 3%), 1.4% of chronic kidney disease^18^ (versus 4%) and 4.2% heart disease^19^(versus 8%). The results were also higher than the 2018 VIGITEL ones, which estimated self-reported prevalence diagnosis of hypertension and diabetes of 25% and 8%, respectively^8^. The higher prevalence of NCDs evidenced here is possibly related to the high percentage of people aged 60 or older in the sample (26%), higher than the percentage of this group among Brazilians in general (14%). As shown in Table 2 and corroborated by other studies, the prevalence of NCDs increases with age^7^.

There are two probable reasons for the higher proportion of older adults in this study compared to the rest of the population. Possibly because of the pandemic, most of the older adults living in the households included in the sample were present at the time of the study, unlike younger people. A population-based study on social distancing patterns conducted in Rio Grande do Sul showed that older adults are more likely to stay home or leave their houses only for essential activities (80%) than individuals aged between 20 and 59 (60%)^20^. Another relevant aspect is that we assessed 133 sentinel cities, which are more populous, have a higher Human Development Index and greater access to health services than the cities excluded from the study. Because of those characteristics, residents of these cities tend to live longer, so the proportion of older adults tends to be higher.

The mask use and physical distancing drastically reduce the transmission of COVID-19^21^. The percentage of participants who reported wearing a mask when leaving home was almost universal in both groups (98%). However, we must consider an information bias when interpreting the results. Knowing the legal requirements of mask use during the pandemic, participants might have reported that they always use masks because this is the correct behavior in the current context.

Carriers of NCDs reported greater adherence to social isolation and universal mask use when leaving home. Nevertheless, the prevalence of antibodies against SARS-CoV-2 in this group was similar to that evidenced among non-carriers of NCDs. This may suggest the occurrence of intra-household transmission of COVID-19, since the proportion of participants who are visited by close relatives and friends was about 50% in both groups. COVID-19 transmission in *clusters*, especially family ones, plays an important role in the rapid spread of the disease^22,23^. The almost universal mask use outside the house is probably rejected at home. Thus, family members who adhere less to isolation and, therefore, present similar infection levels to those evidenced among non-carriers of NCDs would contaminate the carrier ones. However, the adoption of preventive measures such as wearing a mask and physical distancing at home, as well as in-house social distancing, was not evaluated in this study, which prevents us from formally testing this hypothesis. Besides, as previously mentioned, mask use might be overestimated or masks may be worn incorrectly. We must also consider reverse causation. Participants with antibodies against SARS-CoV-2 may have switched to wearing a mask after experiencing COVID-19.

The analysis on the symptom prevalence was restricted to individuals with antibodies against SARS-CoV-2. Among the evaluated COVID-19 symptoms, the prevalence of cough, dyspnea, myalgia and twitching was higher among carriers of NCDs than among non-carriers. Despite the higher prevalence of these symptoms among carriers of NCDs, we must consider an eventual non-responder bias. COVID-19 tends to evolve into severe forms in carriers of NCDs^2^, and severe cases tend to leave after-effects even months after the disease^24^. Therefore, carriers of NCDs possibly developed severe forms of the disease with prolonged after-effects and presented a higher refusal rate, which may have decreased the difference in the symptom prevalence amid groups. In addition, COVID-19 mortality among carriers of NCDs is higher when compared to non-carriers. Hence, participants with a positive result on the rapid test here may represent, to some extent, surviving cases.

This study has other limitations. We assessed data from 133 sentinel cities, which are more developed, have a better health services structure and higher Human Development Index than the cities excluded from the sample. We excluded residents of rural areas, which correspond to 15% of the country’s population. We ignored the adoption of protective behaviors, such as mask use and physical distancing during the interaction with close acquaintances, as well as adherence to distancing recommendations during daily in-house activities. The evaluation of self-reported symptoms may have introduced classification bias. However, we revealed the rapid test results only at the end of the interview, so the participants were blind to them while answering the questionnaire. This possibly decreased the bias likelihood.

The strengths of this study include: its national scope, being one of the world’s largest COVID-19 serological surveys in sample size and territorial coverage carried out so far; the blinding of participants about the test results; and the multidimensional character of the research, which evaluates both seroprevalence and several individual aspects related to COVID-19, such as the presence of comorbidities, adoption of preventive measures against COVID-19 and symptom occurrence.

The results here improve the understanding of the pandemic dynamics in Brazil, where the NCDs burden, as well as incidence and mortality due to COVID-19 are high. Since 2016 Brazil has been suffering from fiscal austerity policies, which constricted the Unified Health System (SUS). Consequently, the supply and quality of actions and services decreased, compromising the health of a large portion of Brazilians who depend on it^25^. Combined with this context, the absence of a national policy to deal with COVID-19 based on scientific evidence^26,27^ contributes substantially to Brazil being placed among the top countries at the global ranking of new infections and deaths from the disease^1^.

The pandemic control in Brazil requires large-scale immunization of the population. Immunization against COVID-19 in Brazil began in mid-January 2021, firstly applied to health professionals working on the front line against the virus. Carriers of NCDs are part of the third priority group at the Brazilian Ministry of Health’s National Immunization Plan^28^. Despite having one of the largest and most efficient immunization networks in the world, vaccination in Brazil is still incipient and the country currently ranks 78th in the world ranking of countries with the highest number of citizens vaccinated against the disease (10.3 /100 inhabitants)^29^. The immunization of NCDs carriers lacks a prediction.

In conclusion, the prevalence of chronic diseases in Brazil is high and the COVID-19 pandemic affects carriers and non-carriers of chronic diseases similarly. Carriers of NCDs present more severe forms of COVID-19 and higher symptom prevalence. Greater adherence to social distancing measures among carriers of NCDs is disassociated from a lower incidence of COVID-19 in this group. More than ever, we must stimulate protective measures such as physical distancing and wearing a mask at home in this group.
