# Supplementary material for: Chronic non-communicable diseases and COVID-19: EPICOVID-19 Brazil results
Source: Rev Saude Publica. 2021 May 21;55:38. doi: 10.11606/s1518-8787.2021055003673 (PMC8139841; doi:10.11606/s1518-8787.2021055003673)
Supplement: Translation

# Translation: Doenças crônicas não transmissíveis e covid-19: resultados do estudo Epicovid-19 Brasil

**Authors:** Marilia Arndt Mesenburg, Pedro Curi Hallal, Ana Maria Baptista Menezes, Aluísio J D Barros, Bernardo Lessa Horta, Fernando Celso de Barros, Fernando Pires Hartwig, Nadège Jacques, Mariangela Freitas da Silveira

**Affiliations:** I Brasil Universidade Federal de Pelotas. Programa de Pós-Graduação em Epidemiologia. Pelotas, RS, Brasil.; II Brasil Universidade Federal de Ciências da Saúde de Porto Alegre. Departamento de Saúde Coletiva. Porto Alegre, RS, Brasil.

**Keywords:** Infecções por Coronavírus, epidemiologia, Doenças não Transmissíveis, Fatores Socioeconômicos, Inquéritos Epidemiológicos

## Abstract

**OBJETIVO:**

Descrever a prevalência de doenças crônicas e fatores socioeconômicos e demográficos associados, avaliar os padrões de distanciamento social e a prevalência de anticorpos contra SARS-CoV-2 e sintomas de covid-19 em portadores e não portadores de doenças crônicas.

**MÉTODOS:**

Foram avaliados dados de 77.075 mil indivíduos de 20 a 59 anos de três etapas do inquérito sorológico de abrangência nacional Epicovid-19 Brasil, realizadas entre maio e junho de 2021. A presença de anticorpos contra SARS-CoV-2 foi avaliada por teste rápido. Foram investigadas as prevalências autorreferidas de hipertensão, diabetes, asma, câncer, doença renal crônica e doença cardíaca. A prevalência de uso de máscara, de adesão a medidas de isolamento e de anticorpos foi avaliada separadamente entre portadores e não portadores de doenças crônicas. A prevalência de sintomas foi avaliada entre doentes crônicos e não doentes portadores de anticorpos.

**RESULTADOS:**

A prevalência do pelo menos uma doença crônica foi de 43%, maior na região Sudeste, entre indivíduos brancos e indígenas, mulheres, menos escolarizados e em menor posição socioeconômica. O uso de máscara ao sair do domicílio não diferiu entre doentes crônicos e não doentes (98%). A proporção de participantes que referiram adesão ao isolamento foi maior entre doentes crônicos (15,9%) que entre não doentes (24,9%). A prevalência de anticorpos contra SARS-CoV-2 foi semelhante entre doentes crônicos e não doentes (2,4% e 2,3%). A prevalência de tosse, dispneia, palpitações e mialgia foi significativamente maior entre doentes crônicos, mas a proporção de sintomáticos não diferiu entre os grupos.

**CONCLUSÃO:**

A prevalência de doenças crônicas no país é alta e a pandemia de covid-19 atinge de forma semelhante doentes e não doentes. Doentes crônicos apresentam formas mais graves de covid-19 e maior prevalência de sintomas. A maior adesão às medidas de distanciamento social entre doentes crônicos não se reflete em menor incidência de covid-19 nesse grupo.

## INTRODUÇÃO

Até o início de abril de 2021, foram contabilizados cerca de 132 milhões de casos confirmados de covid-19 e 3 milhões de óbitos no mundo. As Américas e a Europa contribuem com o maior número de casos confirmados. O Brasil contabiliza aproximadamente 3% da população mundial, mas concentra 10% de todos os casos e mortes do planeta^1^. A mortalidade por covid-19 depende não apenas do número de casos incidentes, mas também de fatores populacionais como estrutura etária e carga de comorbidades relacionadas ao desenvolvimento de formas severas da doença^1^.

Desde o início da pandemia, doenças crônicas não transmissíveis (DCNT) como hipertensão arterial, diabetes, doenças respiratórias e doenças cardiovasculares vêm sendo associadas à maior severidade e letalidade da covid-19^2^. Uma metanálise sobre a relação entre DCNT e severidade de covid-19, incluindo oito estudos, evidenciou que doença pulmonar obstrutiva crônica (DPOC) foi o mais forte preditor de formas severas de covid-19 (*odds ratio* [OR] = 6,4; IC95% 2,4–16,9), seguida de doença cardiovascular (OR = 2,7; IC95% 1,5–4,8) e hipertensão (OR = 1,9; IC95% 1,4–2,7). A internação em unidades de terapia intensiva foi ainda mais fortemente associada a DPOC, doença cardiovascular e hipertensão, com OR de 17,8 (IC95% 6,5–48,2), 4,4 (IC95% 2,6–7,4) e 3,6 (IC95% 2,2–5,9)^4^, respectivamente. Outra metanálise avaliou especificamente a relação de diabetes com severidade ou letalidade da covid-19, encontrando associação positiva (OR 2,16; IC95% 1,74–2,68)^5^. Doença renal crônica, câncer, asma, doenças de fígado e HIV/AIDS também estão relacionados à evolução desfavorável da doença^6^.

O Brasil, devido à transição demográfica e epidemiológica em progresso, apresenta alta carga de DCNT^7,8^. A prevalência de obesidade e inatividade física, importantes fatores de risco para essas morbidades, também é alta^8,9^. Esse cenário é alarmante no contexto brasileiro da pandemia de covid-19, pois aumenta o risco de casos graves em um país com elevada incidência. Portanto, a avaliação do panorama das DCNT associadas à infecção covid-19 no Brasil é fundamental. Este estudo tem como objetivo descrever a prevalência de DCNT e fatores socioeconômicos e demográficos associados, além de avaliar os padrões de distanciamento social e a prevalência de anticorpos contra SARS-CoV-2 entre portadores e não portadores de DCNT, assim como a ocorrência de sintomas entre indivíduos com anticorpos contra SARS-CoV-2 portadores e não portadores de DCNT.

## MÉTODOS

Foram analisados dados do estudo Epicovid-19 Brasil, uma serie de inquéritos sorológicos de abrangência nacional realizados com o objetivo de avaliar a magnitude, evolução e características da pandemia. Para fazer parte do Epicovid-19 Brasil, foi selecionada a cidade mais populosa de cada uma das 133 regiões intermediárias nas quais o país é geograficamente dividido pelo Instituto Brasileiro de Geografia e Estatística (IBGE). Em cada cidade foi realizada amostragem em múltiplos estágios, tendo como unidades amostrais primárias os setores censitários urbanos definidos pelo IBGE, utilizando a malha de 2010. Foram selecionados 25 setores em cada cidade, com probabilidade proporcional ao tamanho. Em cada setor foram selecionados sistematicamente 10 domicílios. Em cada domicílio um morador era sorteado e convidado a participar do estudo. No caso de recusa, um segundo morador era sorteado. Caso o segundo morador também recusasse, os entrevistadores abordavam o domicílio imediatamente ao lado direito. O mesmo procedimento de substituição era adotado caso o domicílio selecionado estivesse desocupado ou não atendesse ao entrevistador. Todas as recusas e substituições foram registradas pela equipe de campo.

Cada participante respondeu a um questionário sobre características socioeconômicas e demográficas, distanciamento social, uso de máscara, presença de sintomas de covid-19 e diagnóstico prévio de DCNT, entre outros. Os participantes foram submetidos ao teste rápido *WONDFO SARS-CoV-2 Antibody Test* (Wondfo Biotech Co., Guangzhou, China), que detecta a presença de anticorpos contra SARS-CoV-2 (IgG e IgM). O teste foi previamente validado pela equipe de pesquisadores responsáveis pelo estudo^10,11^. Os entrevistadores responsáveis pela coleta dos dados receberam treinamento padronizado sobre a aplicação do questionário, realização e leitura do teste rápido. Os testes rápidos positivos e 20% dos negativos foram avaliados por um segundo observador. A metodologia detalhada do estudo Epicovid-19 Brasil foi publicada previamente^12^.

Os dados foram coletados em três etapas do Epicovid-19 Brasil, realizadas de 14 a 21 de maio (n = 25.025), de 04 a 07 de junho (n = 31.165) e de 21 a 24 de junho de 2020 (n = 33.207). As análises deste estudo foram restritas a adultos de 20 anos ou mais.

Para avaliar se eram portadores de doenças crônicas, os participantes foram questionados se já haviam recebido diagnóstico médico de hipertensão, diabetes, asma, câncer, doença renal crônica e cardiopatia (“sim” ou “não”). Os indivíduos que relataram diagnóstico de uma ou mais DCNT foram considerados como portadores de DCNT e serão mencionados dessa forma no texto.

A presença de anticorpos contra SARS-CoV-2 foi avaliada pelo resultado do teste rápido: quando o resultado era positivo, considerou-se que o indivíduo apresentava anticorpos contra o vírus. O uso de máscara foi avaliado pela pergunta “Você usa máscara quando sai de casa?” (“sim”, “não” ou “não sai de casa”). A adesão ao distanciamento social foi avaliada pelas seguintes questões:

- “Com relação ao distanciamento social que está sendo orientado pelas autoridades de saúde, ou seja, ficar em casa e evitar contato com outras pessoas, quanto você acha que está conseguindo fazer?” (“muito pouco”, “pouco”, “mais ou menos”, “bastante” ou “praticamente isolado de todo mundo”);
- “Como tem sido a sua rotina de atividades?” (“fica em casa o tempo todo”, “sai apenas para coisas essenciais, como comprar comida”, “sai de vez em quando para fazer compras e esticar as pernas”, “sai todos os dias para alguma atividade”, “sai todos os dias, o dia todo, para trabalhar ou outra atividade regular”);
- “Pensando na rotina da casa, quem tem entrado na casa?” (“somente os familiares que moram junto, se tiver, e mais ninguém”, “alguns parentes próximos visitam de uma a duas vezes por semana”, “alguns parentes próximos visitam quase que todos os dias”, “amigos, parentes ou outros visitam de uma a duas vezes por semana”, “amigos, parentes ou outros visitam quase todos os dias”).

Para avaliar a presença de sintomas de covid-19, os participantes foram questionados se sobre a ocorrência dos seguintes sintomas: febre, tosse, dor de garganta, alterações de olfato ou paladar, mialgia, tremores, dor de cabeça, dificuldade para respirar, palpitação, diarreia e vômitos. Foram considerados sintomáticos os participantes que relataram a ocorrência de pelo menos um sintoma. O período recordatório das questões sobre sintomas diferiu entre as etapas do Epicovid-19 Brasil. As duas primeiras etapas avaliaram a ocorrência de sintomas nos 15 dias anteriores à entrevista e a terceira etapa avaliou desde março de 2020 até o momento da entrevista. Tendo em vista que anticorpos contra SARS-Cov-2 passam a ser detectáveis no teste rápido cerca de 15 dias após a infecção, a análise sobre ocorrência de sintomas foi restrita aos participantes da terceira etapa.

As variáveis socioeconômicas e demográficas avaliadas foram região geográfica (Norte, Nordeste, Sudeste, Sul ou Centro-Oeste), cor da pele autorreferida (branca, preta, parda, amarela ou indígena), sexo (feminino ou masculino), idade (em categorias) e escolaridade (ensino fundamental, ensino médio ou ensino superior). Para avaliar a posição socioeconômica dos domicílios, foi utilizado o Indicador Econômico Nacional (IEN). Esse índice foi criado pela análise de componentes principais baseada em um conjunto de características e bens do domicílio (computador, acesso à internet, televisão colorida, ar-condicionado, veículos automotores, televisão a cabo, número de cômodos utilizados para dormir e número de banheiros). O primeiro componente foi extraído e dividido em quintis. O primeiro quintil representa os 20% mais pobres e o quinto quintil, os 20% mais ricos^13^.

As análises incluíram a descrição da amostra e o cálculo da prevalência de cada DCNT e da prevalência de portadores de DCNT. A prevalência de portadores de DCNT foi avaliada segundo região, cor da pele, sexo, idade, IEN e escolaridade. A prevalência de uso de máscara e de adesão as medidas de isolamento social bem como a prevalência de indivíduos com anticorpos contra SARS-Cov-2 foram calculadas separadamente para portadores e não portadores de DCNT. Por fim, foi calculada a prevalência de sintomas para covid-19 entre portadores e não portadores de DCNT com anticorpos contra SARS-Cov-2. Para todas as proporções, foram calculados intervalos de confiança de 95%. Valores-p foram obtidos por teste do qui-quadrado para heterogeneidade ou, para variáveis categóricas ordinais, tendência linear, considerando um nível de significância de 5%. Todas as análises foram conduzidas no programa estatístico Stata 16, levando em consideração o efeito de delineamento amostral.

Este estudo respeitou todos os preceitos éticos e legislação que rege a pesquisa com seres humanos e foi aprovado pela Comissão Nacional de Ética em pesquisa (CAAE 30721520.7.1001.5313). Todos os participantes assinaram o termo de consentimento livre e esclarecido.

## RESULTADOS

Foram analisados dados de 77.075 participantes de 20 anos ou mais do estudo Epicovid-19 Brasil. A maioria era do sexo feminino (59%) e cor da pele não branca (62%). Cerca de um quarto era idoso (60 anos ou mais). Cerca de 40% dos participantes referiram ter cursado ensino fundamental (39%) e residir na região. Considerando as três etapas do estudo, a proporção bruta de participantes com anticorpos contra SARS-CoV-2 foi de 2,3% (IC95% 2,3–2,5). Cerca de 45% dos relatou ser portador de uma ou mais DCNT. A DCNT mais prevalente foi hipertensão arterial (30%), seguida de diabetes (13%), asma (9%) e cardiopatia (8%) (Tabela 1).

**Tabela 1:** Características sociodemográficas e prevalência de doenças crônicas não transmissíveis no estudo Epicovid-19 Brasil, etapas 1 a 3 (n = 77.075).

	N	% (IC95%)
Descrição das características da amostra		
Região		
Norte	13.075	16,8 (16,5–17,2)
Nordeste	23.033	29,6 (29,1–30,1)
Sudeste	19.359	25,3 (24,8–25,8)
Sul	13.112	17,3 (16,8–17,8)
Centro-Oeste	8.496	11,0 (10,7–11,4)
Cor da pele		
Branca	28.387	37,8 (37,2–38,3)
Parda	33.732	44,8 (44,4–45,3)
Preta	10.030	13,3 (12,9–13,6)
Amarela	2.132	2,8 (2,7–2,9)
Indígena	1.046	1,4 (1,3–1,5)
Sexo		
Masculino	31.239	40,5 (40,2–40,9)
Feminino	45.836	59,5 (59,1–59,8)
Idade		
20–29	13.796	18,3 (18,0–18,6)
30–39	13.726	18,2 (17,9–18,5)
40–49	14.129	18,5 (18,2–18,8)
50–59	14.273	18,5 (18,2–18,8)
60–69	12.062	15,3 (15,1–15,6)
70–79	9.126	11,1 (10,9–11,4)
IEN (em quintis)		
Mais pobres	18.106	22,7 (22,2–23,2)
2	14.161	18,2 (17,9–18,5)
3	14.748	19,3 (18,9–19,6)
4	14.909	19,7 (19,3–20,0)
Mais ricos	15.143	20,2 (19,6–20,8)
Escolaridade		
Ensino fundamental	30.876	39,5 (38,9–40,1)
Ensino médio	28.315	37,7 (37,3–38,1)
Ensino superior	17.013	22,8 (22,2–23,4)
Anticorpos contra SARS-CoV-2		
Sim	1.824	2,3 (2,2–2,5)
Prevalência de doenças crônicas		
Hipertensão	23.575	30,0 (29,6–30,3)
Diabetes	9.842	12,5 (12,2–12,7)
Asma	7.266	9,4 (9,2–9,6)
Câncer	2.307	3,0 (2,8–3,1)
Doença renal	3.128	4,0 (3,8–4,1)
Cardiopatia	6.354	8,1 (7,9–8,3)
Número de doenças crônicas relatadas		
Nenhuma	42.246	56,2 (55,8–56,6)
Uma	20.521	27,0 (26,6–27,3)
Duas	9.164	11,9 (11,6–12,1)
Três	2.985	3,9 (3,7–4,0)
Quatro ou mais	827	1,1 (1,1–1,2)

IC95%: intervalo de confiança de 95%; IEN: Indicador Econômico Nacional.

A Tabela 2 apresenta a prevalência de uma ou mais DCNT segundo características socioeconômicas e demográficas. A prevalência foi maior nas regiões Sudeste e Sul (48%), entre indígenas (47%), mulheres (47%) e indivíduos com menor escolaridade (57%). A prevalência de uma ou mais DCNT apresentou relação inversa com idade e quintis do IEN. Todas as diferenças foram estatisticamente significativas (valor-p < 0,001).

**Tabela 2:** Prevalência de uma ou mais doenças crônicas não transmissíveis, segundo características socioeconômicas e demográficas, no estudo Epicovid-19 Brasil, etapas 1 a 3 (n = 77.075).

	% (IC95%)
Região	p < 0,001^a^
Norte	37,6 (36,7–38,4)
Nordeste	42,2 (41,5–42,9)
Sudeste	48,4 (47,6–49,2)
Sul	47,9 (46,9–48,9)
Centro-Oeste	44,8 (43,6–45,9)
Cor da pele	p < 0,001^a^
Branca	46,9 (46,3–47,5)
Parda	41,4 (40,9–42,0)
Preta	44,3 (43,2–45,3)
Amarela	42,8 (40,7–44,9)
Indígena	47,2 (44,1–50,3)
Sexo	p < 0,001^a^
Masculino	39,5 (38,9–40,1)
Feminino	47,4 (47,0–47,9)
Idade	p < 0,001^b^
20–29	18,8 (18,2–19,5)
30–39	23,6 (22,9–24,3)
40–49	36,7 (35,8–37,5)
50–59	53,9 (53,1–54,8)
60–69	69,0 (68,1–69,8)
70 anos ou mais	78,3 (77,5–79,2)
IEN (em quintis)	p < 0,001^b^
Mais pobres	47,7 (46,9–48,4)
2	45,3 (44,5–46,2)
3	43,3 (42,5–44,1)
4	42,1 (41,3–43,0)
Mais ricos	42,1 (41,3–42,9)
Escolaridade	p < 0,001^b^
Ensino fundamental	57,0 (56,4–57,6)
Ensino médio	35,6 (35,0–36,2)
Ensino superior	35,0 (34,2–35,7)

IC95%: intervalo de confiança de 95%; IEN: Indicador Econômico Nacional.

^a^ Teste do qui-quadrado de heterogeneidade.

^b^ Teste de tendência linear.

Os resultados sobre distanciamento social são apresentados na Tabela 3. Cerca de 25% dos indivíduos portadores de uma ou mais DCNT referiram estar isolados de praticamente todo mundo. Entre os não portadores, esse percentual foi de 15%. Com relação à rotina de atividades, 13% dos portadores de uma ou mais DCNT referiram sair de casa todos os dias para trabalhar, enquanto entre os não portadores de DCNT essa proporção foi de 25%. Tanto entre não portadores quanto entre portadores, a proporção que referiu que somente os próprios moradores frequentam o domicílio foi de aproximadamente 50%. Maior proporção de portadores de uma ou mais DCNT referiu receber parentes próximos (44%) em comparação a não portadores (39%).

**Tabela 3:** Distanciamento social e uso de máscara entre portadores e não portadores de doenças crônicas não transmissíveis (DCNT) no estudo Epicovid-19 Brasil, etapas 1 a 3 (n = 77.075).

	n	Não portador de DCNT	n	Portador de uma ou mais DCNT	p^a^
	
		% (IC95%)		% (IC95%)	
Adesão ao isolamento					
Muito pouca	3.740	8,9 (8,6–9,2)	2.112	6,3 (6,1–6,6)	< 0,001
Pouca	4.449	10,6 (10,3–10,9)	2.621	7,9 (7,6–8,2)	
Mais ou menos	11.637	27,7 (27,2–28,1)	7.533	22,6 (22,2–23,1)	
Bastante	15.510	36,9 (36,4–37,4)	12.706	38,2 (37,6–38,8)	
Isolado	6.686	15,9 (15,5–16,3)	8.300	24,9 (24,5–25,4)	
Rotina de atividades					
Fica em casa o tempo todo	4.983	11,8 (11,5–12,2)	7.930	23,7 (23,3–24,2)	< 0,001
Sai apenas para coisas essenciais como comprar comida	19.676	46,7 (46,2–47,2)	16.482	49,3 (48,8–49,9)	
Sai de vez em quando para compras e esticar as pernas	4.144	9,8 (9,5–10,1)	3.114	9,3 (9,0–9,7)	
Sai todos os dias para alguma atividade	2.884	6,8 (6,6–7,1)	1.546	4,6 (4,4–4,9)	
Sai todos os dias, o dia todo, para trabalhar ou outra atividade regular	10.440	24,8 (24,3–25,3)	4.341	13,0 (12,6–13,4)	
Pessoas que frequentam a casa					
Só os familiares que moram junto, se tiver, e mais ninguém	20.903	49,9 (49,3–50,5)	15.354	46,3 (45,6–46,9)	< 0,001
Alguns parentes próximos visitam uma a duas vezes por semana	13.349	31,9 (31,4–32,4)	11.585	34,9 (34,4–35,5)	
Alguns parentes próximos visitam quase que todos os dias	2.993	7,1 (6,9–7,4)	3.064	9,2 (8,9–9,6)	
Parentes ou outras pessoas visitam uma a duas vezes por semana	2.895	6,9 (6,7–7,2)	1.978	6,0 (5,7–6,2)	
Parentes ou outras pessoas visitam quase todos os dias	1.738	4,2 (3,9–4,4)	1.208	3,6 (3,4–3,9)	
Uso de máscara fora de casa	41.341	98,0 (97,8–98,1)	32.537	97,2 (97,0–97,3)	< 0,001

IC95%: intervalo de confiança de 95%.

^a^ Teste do qui-quadrado de heterogeneidade.

Com exceção dos indivíduos que relataram diagnóstico de câncer, as diferenças na prevalência de anticorpos contra SARS-CoV-2 entre portadores de DCNT e não portadores foram mínimas (Tabela 4). Já a Tabela 5 apresenta a prevalência de sintomas de covid-19 entre indivíduos com anticorpos contra SARS-CoV-2, não portadores e portadores de DCNT. Portadores de uma ou mais DCNT relataram ocorrência de tremores 50% maior que não doentes, além de ocorrência de tosse e dispneia 26% e 29% maior, respectivamente. Mialgia foi 16% mais prevalente entre indivíduos com DCNT. Não houve diferença estatisticamente significativa na prevalência dos demais sintomas avaliados e na proporção de indivíduos assintomáticos.

**Tabela 4:** Prevalência de anticorpos contra SARS-CoV-2 entre portadores de doenças crônicas não transmissíveis (DCNT) e não portadores no estudo Epicovid-19 Brasil, etapas 1 a 3.

DCNT	Não		Sim		RP (IC95%)^a^	p
	
	n	% (IC95%)	n	% (IC95%)		
Hipertensão	1.263	2,4 (2,2–2,5)	552	2,3 (2,1–2,6)	1,0 (0,9–1,1)	0,797
Diabetes	1.542	2,3 (2,2–2,4)	265	2,7 (2,4–3,0)	1,2 (1,0–1,3)	0,019
Asma	1.664	2,4 (2,3–2,5)	154	2,1 (1,8–2,5)	0,9 (0,8–1,0)	0,148
Câncer	1.794	2,4 (2,3–2,5)	22	1,0 (0,6–1,4)	0,4 (0,3–0,6)	< 0,001
Doença renal	1.732	2,4 (2,2–2,5)	82	2,6 (2,1–3,3)	1,1 (0,9–1,4)	0,332
Cardiopatia	1.710	2,4 (2,3–2,6)	101	1,6 (1,3–1,9)	0,7 (0,5–0,8)	< 0,001
Uma ou mais DCNT	1.009	2,4 (2,2–2,6)	769	2,3 (2,1–2,5)	1,0 (0,9–1,1)	0,403

IC95%: intervalo de confiança de 95%; RP: razão de prevalência.

^a^ Referência: não portadores de doenças crônicas.

**Tabela 5:** Prevalência de sintomas autorreferidos entre indivíduos com anticorpos contra SARS-CoV-2 portadores e não portadores de doenças crônicas não transmissíveis (DCNT) no estudo Epicovid-19 Brasil, etapa 3.

Sintoma	Não portadores de DCNT		Portadores de DCNT		RP (IC95%)^a^	p^b^
	
	n	% (IC95%)	n	% (IC95%)		
Febre	242	50,8 (45,6–56,0)	196	55,2 (49,3–61,0)	0,9 (0,9–1,2)	0,212
Dor de garganta	174	36,6 (31,9–41,5)	124	34,9 (29,6–40,6)	1,0 (0,8–1,2)	0,629
Tosse	214	45,1 (40,2–50,0)	202	56,6 (50,7–62,3)	1,3 (1,1–1,5)	0,001
Dispneia	111	23,4 (19,4–27,8)	107	30,1 (24,8–35,9)	1,3 (1,0–1,6)	0,030
Palpitações	80	16,9 (13,4–21,1)	103	29,1 (24,0–34,8)	1,7 (1,3–2,3)	0,381
Anosmia	285	60,0 (55,0–64,8)	223	63,0 (57,3–68,3)	1,1 (0,9–1,2)	0,381
Diarreia	137	28,8 (24,3–33,7)	108	30,3 (25,1–36,0)	1,1 (0.8–1,3)	0,645
Vomito	36	7,6 (5,4–10,6)	36	10,1 (6,9–14,4)	1,3 (0,8–2,2)	0,203
Mialgia	233	46,9 (42,2–51,8)	192	54,4 (48,4–60,3)	1,2 (1,0–1,3)	0,034
Tremores	90	19,1 (15,2–23,7)	104	29,2 (24,3–34,7)	1,5 (1,1–2,0)	0,001
Dor de cabeça	295	62,0 (57,0–66,7)	202	56,6 (50,8–62,2)	0,9 (0,8–1,0)	0,116
Sintomáticos	403	86,9 (83,2–89,8)	313	90,0 (86,5–94,1)	1,1 (1,0–1,1)	0,067

IC95%: intervalo de confiança de 95%; RP: razão de prevalência.

^a^ Referência: não portadores de doenças crônicas.

^b^ Teste do qui-quadrado de heterogeneidade.

## DISCUSSÃO

Os resultados deste estudo evidenciaram que o Brasil apresenta alta carga de doenças crônicas. Apesar da maior adesão às medidas de distanciamento social entre portadores de DCNT, a prevalência de anticorpos contra SARS-CoV-2 não diferiu entre os grupos.

A prevalência de cada morbidade avaliada neste estudo foi maior em comparação aos resultados da Pesquisa Nacional de Saúde, na qual 21% dos participantes referiam diagnóstico de hipertensão^14^ (versus 30% no presente estudo), 6,2% de diabetes^15^ (versus 13%), 4,4% de asma^16^ (versus 9%), 1,8% de câncer^17^ (versus 3%), 1,4% de doença renal crônica^18^ (versus 4%) e 4,2% de cardiopatia^19^(versus 8%). Os resultados também foram superiores ao estudo Vigitel 2018, que estimou prevalências de diagnóstico de hipertensão e diabetes autorreferidas de 25% e 8%, respectivamente^8^. É possível que a maior prevalência de DCNT evidenciada neste estudo seja em decorrência do percentual elevado do pessoas com 60 anos ou mais na amostra (26%), superior ao percentual desse grupo na população geral brasileira (14%). Como demonstrado na Tabela 2 e corroborado por outros estudos, a prevalência de DCNT aumenta com a idade^7^.

A maior proporção de idosos neste estudo em comparação ao Brasil é, provavelmente, decorrente de duas situações. Possivelmente por causa da pandemia, a maior parte dos idosos residentes nos domicílios incluídos na amostra estava presente no momento da realização do estudo, diferentemente dos jovens. Um estudo de base populacional sobre padrões de distanciamento social realizado no Rio Grande do Sul evidenciou que a proporção de idosos que não sai do domicílio ou sai apenas para atividades essenciais é superior à de indivíduos entre 20 e 59 anos (cerca de 80% e 60%, respectivamente)^20^. Outro aspecto a ser considerado é que o estudo foi realizado em 133 cidades sentinelas, que são mais populosas, apresentam maior índice de desenvolvimento humano e maior acesso a serviços de saúde que as cidades não incluídas no estudo. Por apresentarem essas características, residentes dessas cidades tentem a ter maior expectativa de vida e a proporção de idosos tende a ser maior.

O uso de máscara e distanciamento físico reduzem drasticamente a transmissão da covid-19^21^. O percentual de participantes que referiu uso de máscara sempre que sai de casa foi quase universal em ambos os grupos (98%). Entretanto, ao interpretar esse resultado, é necessário considerar a presença de viés de informação. É possível que, sabendo que o uso de máscara é recomendado ou exigido por lei durante a pandemia, os participantes tenham relatado que utilizam máscara sempre porque esse é o comportamento correto no contexto atual.

Portadores de DCNT referiram maior adesão ao isolamento social e uso universal de máscara ao sair de casa. Entretanto, a prevalência de anticorpos contra SARS-CoV-2 nesse grupo não diferiu daquela evidenciada entre não portadores de DCNT, o que pode sugerir a ocorrência de transmissão intradomiciliar de covid-19, já que a proporção de participantes que recebe visita de familiares e amigos próximos foi cerca de 50% nos dois grupos. A transmissão da covid-19 em *clusters*, especialmente familiares, desempenha um papel importante na rápida propagação da doença^22,23^. É possível que o uso de máscara, quase universal fora do domicílio, não seja adotado em casa no convívio familiar, o que faria com que portadores de DCNT fossem contaminados por familiares que aderem menos ao isolamento e, portanto, apresentassem níveis semelhantes de infecção aos evidenciados entre não portadores de DCNT. Entretanto, a adoção de medidas de prevenção como o uso de máscara e o distanciamento físico durante o convívio familiar, bem como o distanciamento social por parte dos demais residentes do domicílio, não foi avaliado neste estudo, o que nos impede de testar formalmente essa hipótese. Além disso, conforme mencionado anteriormente, é possível que o uso de máscara esteja superestimado ou que a máscara não seja utilizada de forma adequada. Também é preciso considerar a possibilidade de causalidade reversa. Participantes com anticorpos contra SARS-CoV-2 podem ter passado a utilizar máscara após terem tido covid-19.

A análise sobre prevalência de sintomas foi restrita a indivíduos com anticorpos contra SARS-CoV-2. Dentre os sintomas de covid-19 avaliados, a prevalência de tosse, dispneia, mialgia e tremores foi maior entre portadores de DCNT, em comparação a não portadores. Apesar da maior prevalência desses sintomas entre portadores de DCNT, é importante considerar a possibilidade de viés de não respondentes. Portadores de DCNT tendem a evoluir para formas graves de covid-19^2^, e casos graves tendem a apresentar sequelas mesmo meses após a doença^24^. Portanto, é possível que portadores de DCNT tenham desenvolvido formas graves da doença com sequelas por período prolongado e tenham apresentado maior taxa de recusa, o que pode ter diminuído a diferença na prevalência de sintomas entre os grupos. Além disso, a mortalidade por covid-19 entre portadores de DCNT é maior quando comparada a não portadores. Portanto, os participantes com resultado de teste rápido positivo captados pelo estudo podem representar, em alguma medida, casos sobreviventes.

Este estudo apresenta ainda outras limitações. A coleta de dados foi realizada em 133 cidades sentinelas, que são mais desenvolvidas, possuem melhor estrutura dos serviços de saúde e maior índice de desenvolvimento humano que as cidades não incluídas na amostra. Residentes em zonas rurais, que correspondem a 15% da população do país, não foram incluídos no estudo. Não foram avaliadas a adoção de comportamentos protetores, como o uso de máscara e distanciamento físico, durante o convívio com pessoas próximas, e a adesão às recomendações de distanciamento por outros residentes do mesmo domicílio. A avaliação de sintomas autorreferidos pode ter introduzido viés de classificação. Entretanto, enquanto respondiam ao questionário, os participantes eram cegos em relação ao resultado do teste rápido, revelado ao final da entrevista, o que possivelmente diminuiu a probabilidade do viés.

Como pontos fortes destacam-se a abrangência nacional do estudo, que é, em nível mundial, um dos maiores inquéritos sorológicos sobre covid-19 em tamanho amostral e cobertura territorial realizados até agora; o cegamento dos participantes sobre o resultado do teste; e o caráter multidimensional da pesquisa, que avalia não só a soroprevalência, mas diversos aspectos individuais relacionados à covid-19, como presença de comorbidades, adoção de medidas preventivas contra covid-19 e ocorrência de sintomas.

Os resultados deste estudo contribuem para a compreensão da dinâmica da pandemia no Brasil, que apresenta alta carga de DCNT e alta incidência e mortalidade por covid-19. O país, desde 2016, vem sofrendo com políticas de austeridade fiscal, que levaram ao encolhimento do Sistema Único de Saúde (SUS), consequente diminuição da oferta e qualidade das ações e serviços, e à degradação da saúde de grande parcela da população brasileira que dele depende^25^. Aliada a esse contexto, a ausência de uma política nacional de enfrentamento à covid-19 baseada em evidências científicas^26,27^ contribui substancialmente para que o Brasil ocupe um dos primeiros lugares no ranking mundial de novas infecções e mortes pela doença^1^.

O controle da pandemia no Brasil requer a imunização em larga escala da população. A imunização contra covid-19 no Brasil teve início em meados de janeiro de 2021, sendo aplicada primeiramente a profissionais da saúde que atuam na linha de frente contra a covid-19. Os portadores de doenças crônicas fazem parte do terceiro grupo prioritário no Plano Nacional de Imunização do Ministério da Saúde do Brasil^28^. A vacinação no país ainda é incipiente e, apesar de apresentar uma das maiores e mais eficientes redes de imunização do mundo, o Brasil ocupa atualmente a 78ª posição no ranking mundial de países com maior número de vacinados contra a doença (10,3 /100 habitantes)^29^. Ainda não há previsão de quando portadores de DCNT serão imunizados.

Em conclusão, a prevalência de DCNT no país é alta e a pandemia de covid-19 atinge de forma semelhante portadores e não portadores delas. Portadores de DCNT apresentam formas mais graves de covid-19 e maior prevalência de sintomas. A maior adesão às medidas de distanciamento social entre portadores de DCNT não se reflete em menor incidência de covid-19 nesse grupo. Mais do que nunca, é fundamental que medidas de proteção como distanciamento físico e uso de máscara durante o convívio domiciliar sejam estimuladas nesse grupo.
